# Surgical Restoration of Antero-Apical Left Ventricular Aneurysms: Cardiac Computed Tomography for Therapy Planning

**DOI:** 10.3389/fcvm.2022.763073

**Published:** 2022-03-28

**Authors:** Natalia Solowjowa, Olena Nemchyna, Yuriy Hrytsyna, Alexander Meyer, Felix Hennig, Volkmar Falk, Christoph Knosalla

**Affiliations:** ^1^Department of Cardiothoracic and Vascular Surgery, German Heart Center Berlin, Berlin, Germany; ^2^DZHK (German Centre for Cardiovascular Research), Partner Site Berlin, Berlin, Germany; ^3^Charité – Universitätsmedizin Berlin, Berlin, Germany; ^4^Eidgenössiche Technische Hochschule Zürich, Department of Health Sciences and Technology, Translational Cardiovascular Technology, Zurich, Switzerland

**Keywords:** heart failure, myocardial infarction, aneurysm, imaging—computed tomography, cardiac computed tomography, surgical ventricular reconstruction

## Abstract

**Background:**

Surgical ventricular restoration (SVR) leads to functional improvement by volume reduction and restoration of left ventricular (LV) geometry. Our purpose was to refine the planning for SVR using cardiac computed tomography (CCT).

**Methods:**

The possibility to anticipate the postoperative residual LV volume was assessed using CCT in 205 patients undergoing SVR combined with coronary artery bypass grafting (77%), mitral valve repair/replacement (19%) and LV thrombectomy (19%). The potential of CCT to guide the procedure was evaluated. Additionally, the predictive value of CCT characteristics on survival was addressed.

**Results:**

30-day, 1- and 5-year survival was 92.6, 82.7, and 72.1%, respectively, with a marked reduction of NYHA class III-IV quota after surgery (95.1% vs. 20.5% in the follow-up). Both pre- and postoperative LV end-systolic volume index (LVESVI) were predictive of all defined endpoints according to the following tertiles: preoperative: <74 ml/m^2^, 74–114 ml/m^2^ and >114 ml/m^2^; postoperative: <58 ml/m^2^, 58–82 ml/m^2^ and >82 ml/m^2^. On average, a 50 ml/m^2^ increase of preoperative LVESVI was associated with a 35% higher hazard of death (*p* = 0.043). Aneurysms limited to seven antero-apical segments (1–7) were associated with a lower death risk (*n* = 60, HR 0.52, CI 0.28–0.96, *p* = 0.038). LVESVI predicted by CCT was found to correlate significantly with effectively achieved LVESVI (*r* = 0.87 and *r* = 0.88, respectively, *p* < 0.0001).

**Conclusions:**

CCT-guided SVR can be performed with good mid-term survival and significant improvement in HF severity. CCT-based assessment of achievable postoperative LV volume helps estimate the probability of therapeutic success in individual patients.

## Introduction

Chronic ischemic heart failure (HF) after myocardial infarction (MI) is an important indicator of poor prognosis despite advances in drug and device therapy (8). The development of therapeutic concepts addressing ischemic left ventricular (LV) remodeling due to scar formation is essential to improve survival and amelioration of HF symptoms in this patient cohort.

Surgical ventricular restoration (SVR) of anterior LV aneurysms (9–11) is an established treatment option for LV remodeling after MI. It can be performed, if required, in combination with revascularization and mitral valve (MV) repair/replacement (1, 12, 13). Despite disillusioning reports from the STICH trial (2) with its known methodological limitations (14), other studies report excellent outcomes for acute reverse remodeling following SVR. Essential factors of clinical success are appropriate patient selection (15, 16) combined with adequate surgical volume reduction and reshaping of the LV (3, 17).

The analysis of LV volume, scar localization and extension as well as the amount of geometrical distortion play a key role in the preoperative assessment for SVR. Image guidance and a tailored surgical procedure are of paramount importance for a successful SVR. With its high spatial resolution of up to 0.4 mm and an acceptable time resolution of up to 75 ms, cardiac computed tomography (CCT) enables the analysis of a primarily acquired three-dimensional data set, the detailed examination of the cardiac anatomy and the assessment of the functional and geometrical remodeling of the cardiac chambers, based on exact true volume detection, with very high accuracy (4). Advanced CCT software tools allow for analyzing LV segmental wall motion abnormalities (WMA) and estimating the aneurysm volume.

This study aimed to refine surgical planning for SVR and to investigate factors that allow for predicting whether the therapeutic goals (sufficient volume reduction, sufficient residual volume, improved ventricular geometry) can be successfully achieved using the volumetric, geometric and functional tools of CCT. We analyzed our single-center early and mid-term results and the factors determining survival and improvement in HF symptoms after CCT-guided SVR for anterior LV aneurysms.

## Materials and Methods

### Study Design

Using a modified Dor technique in most cases, SVR was performed in patients with severe HF symptoms, well-defined antero-septal LV akinesia or dyskinesia and preserved contractility of residual myocardium after anterior MI. The procedure was combined with coronary revascularization, MV repair or replacement and LV thrombectomy, when indicated. Endpoints were 30-day mortality, death from any cause in the follow-up period, implantation of a LV assist device and heart transplantation.

The diagnosis of LV aneurysm was made by echocardiography or angiography and confirmed by CCT. Echocardiography and CCT were repeated during the first postoperative week. The pre- and postoperative data were analyzed and compared retrospectively. Clinical, demographic and procedural data were retrospectively collected.

### Study Population

We retrospectively analyzed the data of 205 consecutive patients with coronary artery disease who underwent SVR for anterior LV aneurysms in our hospital between November 2005 and January 2016. All patients had a previous anterior MI with severe LV systolic dysfunction and symptomatic HF.

Written informed consent for surgery was obtained from all patients or their representatives. The study was performed according to the principles of the Declaration of Helsinki and approved by the ethics committee of the Charité – Universitätsmedizin Berlin (EA2/177/20).

### Surgical Technique

All SVR procedures were performed through a median sternotomy approach under cardiopulmonary bypass (CPB). After coronary artery bypass grafting (CABG), LV repair and MV repair or replacement were performed. Antero-apical SVR was routinely performed using a modified Dor procedure with several Fontan sutures along the aneurysm perimeter without patch application to exclude the aneurysm and restore LV geometry (9). This technique enables the effective exclusion of scarred area, achieving the required target LV volume and reconstruction of the LV apex. Only few patients (12.2%) with specific local findings, for example a ventricular septal defect after a huge antero-apical MI, required a patch repair.

Intraoperative transesophageal echocardiography was routinely performed to assess the adequacy of LV repair. Mitral surgery was performed in patients with echocardiographically proven MR grade ≥2. All surgeons had access to the complete preoperative CCT assessment.

### MSCT Measurements

#### LV Volumetric Study

CCT measurements have been extensively described previously (5). The data were obtained and analyzed in a single lab by use of the uniform protocols. In brief, contrast-enhanced ECG-triggered cardiac scanning was performed using a first- or second-generation dual-source scanner (Somatom Definition, Somatom Definition Flash, Siemens AG, Erlangen, Germany). To study the anatomy and geometry of the LV, the data set was reconstructed with a slice thickness of 0.75 mm and reconstruction increment of 0.4 mm, from early systole to end diastole in steps of 10%. LV volumes and systolic function were assessed using a dedicated CCT evaluation software (syngo.via Cardiac Function, Siemens AG) and applying a 3D threshold segmentation algorithm. The timepoints of the end diastole and end systole were estimated automatically and adjusted by the investigator if required.

**Figure 1:**
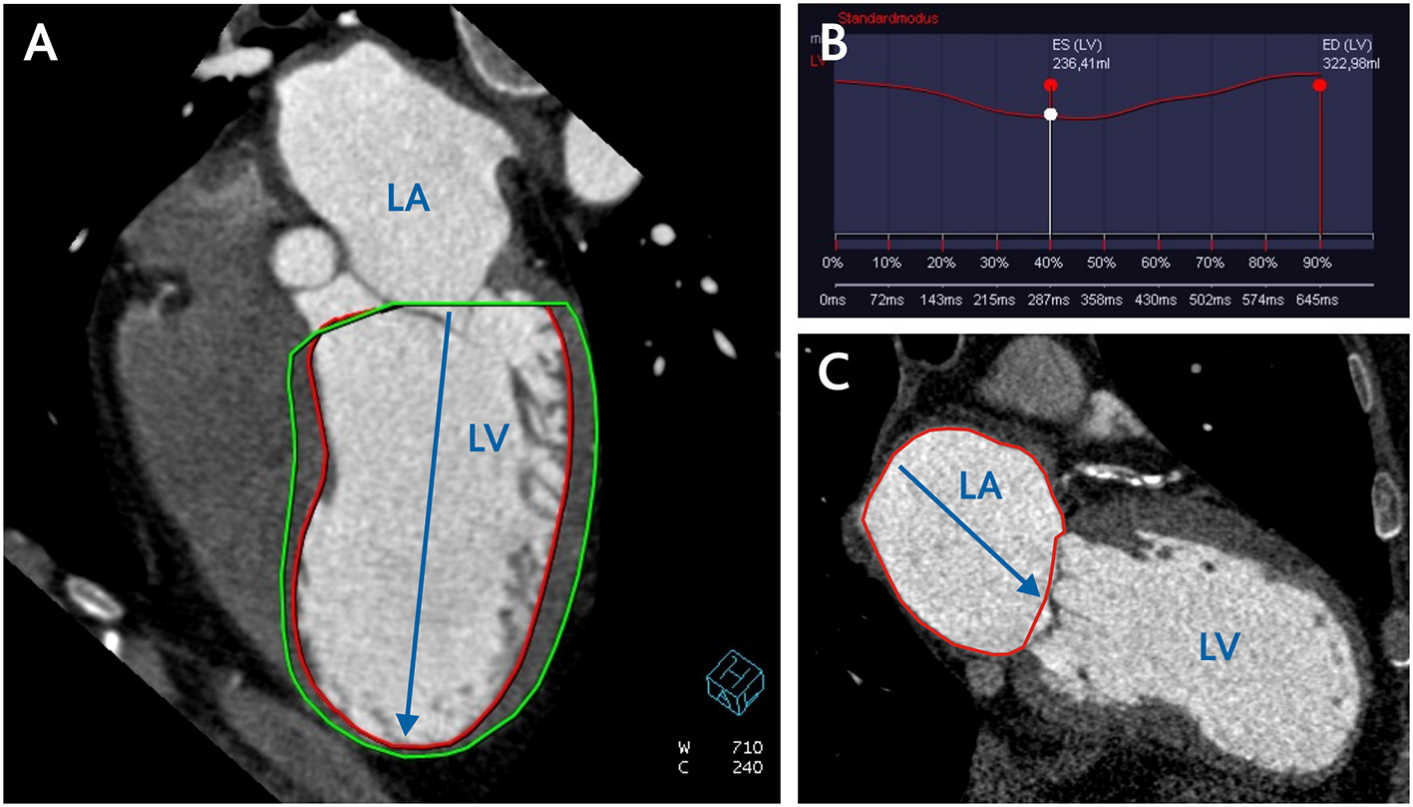
CCT assessment of left ventricular and left atrial volume. **(A)** CCT reconstruction of four-chamber view for semi-automatic measurement of LV volume and calculation of LV volumetric sphericity index (LVSI) according to the formula LVSI = LV Volume/LV long axis 3 × π/6. **(B)** filling curve of LV. **(C)** two-chamber view for measurement of LA area and calculation of LA volume according to the formula (0.85 × A1 × A2)/L. LV, left ventricle; LA, left atrium; A1 and A2, LA area in two- and four-chamber view; L, length of the left atrium.

LV end-diastolic and end-systolic volume (LVEDV, LVESV) were obtained (Figure 1) and then indexed to body surface area (LVEDVI/LVESVI). Stroke volume (SV) was calculated by subtracting LVESV from LVEDV; left ventricular ejection fraction (LVEF) was obtained by dividing SV by LVEDV; cardiac output (CO) was calculated as a product of SV and cardiac rate.

#### Assessment of the Anticipated Residual LV Volume and Aneurysm Volume

**Figure 2:**
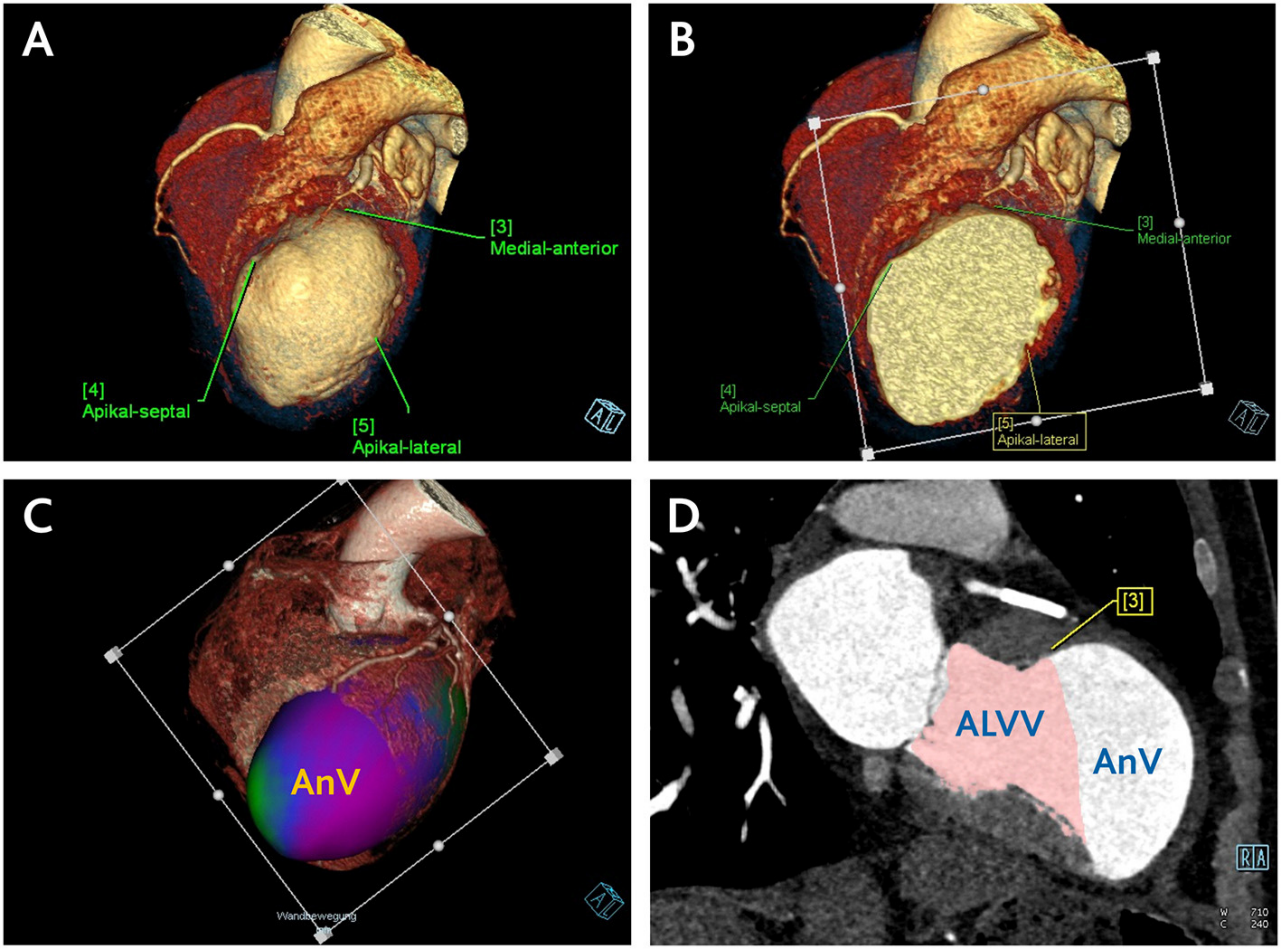
Central Picture. CCT assessment of anticipated residual LV volume and aneurysm volume. **(A)** Definition of three landmarks on the borders of scarred to intact LV myocardium. **(B)** Positioning of the plane determined by the defined landmarks. **(C)** Separation of the aneurysm volume in a three-dimensional data set using the defined plane in the systole and diastole. **(D)** Estimation of the anticipated postoperative LV volume by means of manually corrected LV borders along the defined plane using the same CCT volumetric software tool. Subsequent calculation of the aneurysm volume through subtraction of anticipated from original LV volume. ALVV, anticipated left ventricular volume; AnV, aneurysm volume.

To separate the aneurysm volume in diastole and systole, a plane determined by three landmarks on the borders of scarred to intact LV myocardium (antero-septal, lateral and inferior) was used. The residual volumes were then assessed using the same CCT evaluation software tool (Figure 2). This enabled the estimation of the anticipated postoperative LVEDV/LVEDVI and LVESV/LVESVI. Aneurysm volume (AnV/AnVI) was then calculated as the subtraction of anticipated from original volumes.

#### Analysis of LV Segmental WMA

Local WMA were estimated semiquantitatively using a 17-segment model with separate assessment of papillary muscles. Akinetic or dyskinetic segments involved in an aneurysm or scar formation of other localizations were detected.

Automated cluster analysis was performed to establish the different scar patterns. The identified clusters were then tested for their influence on survival after SVR.

Patients were divided into defined subgroups according to their LVA localization and extension: antero-apical LVA (segments 7, 8, 13, 14, 15, 16, 17)—subgroup1; antero-apical and basal septal or basal anterior LVA (plus segments 1 or 9)—subgroup 2; antero-apical LVA and additional scar of any other localization—subgroup 3.

Morphological characteristics of the aneurysm in terms of myocardial disruption, apposition of thrombotic masses and pericardial effusion were also evaluated.

#### LV Volumetric Sphericity Index

The systolic and diastolic LV volumetric sphericity index (LVSI) was calculated on the basis of end-diastolic and end-systolic LV volume and LV long axis length in two-chamber view according to the empiric formula (7) (Figure 1),


$$\begin{array}{l} \text{LVSI}=\text{LV volume}/\text{LV long axi}{\text{s}}^{3}\times \;\pi /6. \end{array}$$

#### Left Atrium Volume

The left atrium (LA) volume was calculated at the end systole on the basis of planimetric measured LA area in two-chamber view (A1) and four-chamber view (A2) and LA length (L) according to the simplified empiric formula (6) (Figure 1),


$$\begin{array}{l} \left(0.85\times \text{A1}\times \;\text{A2}\right)/\text{L}. \end{array}$$

### Follow-Up Data Collection

Follow-up was performed in 94.6% of the cases, either during a routine clinical evaluation in our outpatient department or by telephone contact using the short form Health Survey (SF-12) (18). In patients, in whom the follow-up was not possible, national death registry check was performed. Mean follow-up time in the overall population was 1,600 ± 1,106-days. Median follow-up time was 1,528-days.

### Statistical Analysis

Continuous variables are presented as mean ± SD. Parametric and non-parametric tests were used where required. For parametric variables, means were compared with the paired, two-tailed student *t*-test. Categorical variables are presented as numbers with percentages and compared using chi-square tests. Actuarial survival curves were produced using the Kaplan-Meier analysis with a long-rank test. For the evaluation of survival differences dependent on LV volumetric parameters, the patient population was subgrouped according to tertiles of LVESVI with further application of survival analysis. A cluster analysis of scarred ventricle segments detected in CCT was performed using nearest neighbor algorithm to reveal patterns of segmental wall motion abnormalities associated with better or worse survival.

Cox proportional hazard regression modeling was applied, and both univariable and multivariable hazard ratios were calculated to assess independent predictive factors of combined endpoint of all-cause mortality, implantation of a LV assist device and heart transplantation. A *p-*value <0.05 was considered statistically significant. The data were analyzed with SPSS 23 (SPSS, Chicago, IL, USA).

## Results

Preoperative patient characteristics are presented in Table 1. There were 10 (4.9%) patients in New York Heart Association (NYHA) class II of HF, 179 (87.3%) in class III and 16 in class IV (7.8%); the mean NYHA class was 3.02 ± 0.35. The median preoperative LVEF, estimated via echocardiography, was 30% (11–70). 36 (17.6%) patients had a concomitant MR of grade ≥2.

**Table 1:** Baseline patient characteristics and perioperative data.

**Variable**	**All patients (*n =* 205)**
Mean age, y	63.4 ± 11.2
Male sex	151 (73.6%)
Diabetes mellitus	62 (30.2%)
Arterial hypertension	140 (68.3%)
Hypercholesterolemia	139 (67.8%)
Peripheral artery disease	20 (9.7%)
Renal failure	32 (15.6%)
Atrial fibrillation	23 (11.2%)
**NYHA class**	
II	10 (4.9%)
III	179 (87.3%)
IV	16 (7.8%)
Median LV EF (%, Echo) (range)	30 (11–70)
Mitral regurgitation ≥2+	36 (17.6%)
**Coronary lesion**	
Single vessel	34 (16.6%)
Double vessel	43 (21%)
Triple vessel	112 (54.6%)
No lesion	16 (7.8%)
**Perioperative data**	
Concomitant CABG	158 (77.1%)
Median no. of grafts performed (range)	2 (0–5)
Concomitant MV repair/replacement	39 (35/4) (19%)
Linear repair	180 (87.8%)
Patch repair	25 (12.2%)
LV thrombectomy	39 (19%)
Median CBP time, min (range)	131 (40–693)
Mean cross-clamp time, min	79.7 ± 35
IABP support	38 (18.5%)
Switch to LVAD	7 (3.4%)
**Postoperative complications**	
Reexploration for bleeding	15 (7.3%)
Stroke	1 (0.4%)
Sepsis	8 (3.9%)
Renal failure	6 (2.9%)
Hospital (30-day) mortality	15 (7.3%)

A modified Dor procedure without patch application was performed in 180 patients (87.8%). while 25 patients underwent SVR with patch. One hundred and fifty eight patients (77.1%) had associated myocardial revascularization. Thirty nine patients (19%) underwent concomitant MV surgery (MV repair in 35 and MV replacement in four patients). LV thrombectomy was performed in 39 patients (19%).

Fifteen patients (7.3%) died in hospital within 30-days due to multiorgan failure (*n* = 9), septic shock (*n* = 4), electromechanical dissociation (*n* = 1) or stroke (*n* = 1). The operative and postoperative data are summarized in Table 1.

### Early and Late Outcomes

**Figure 3:**
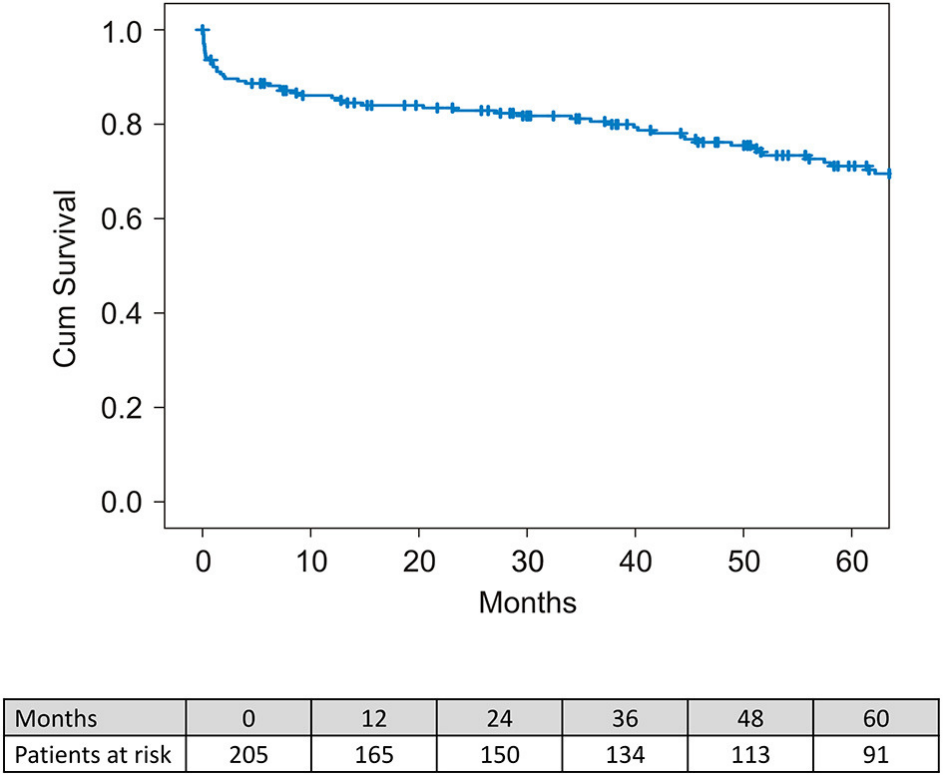
Kaplan-Meier survival curve for all-cause mortality for the overall population.

The median survival period was 51.1 months (range between 1-day and 121.4 months) and the mean survival time was 51.6 ± 37.8 months. Overall, 30-day mortality was 7.3% (*n* = 15). One- and 5-year survival was 82.7 and 72.1%, respectively (Figure 3).

### NYHA Class Change in Survivors

**Figure 4:**
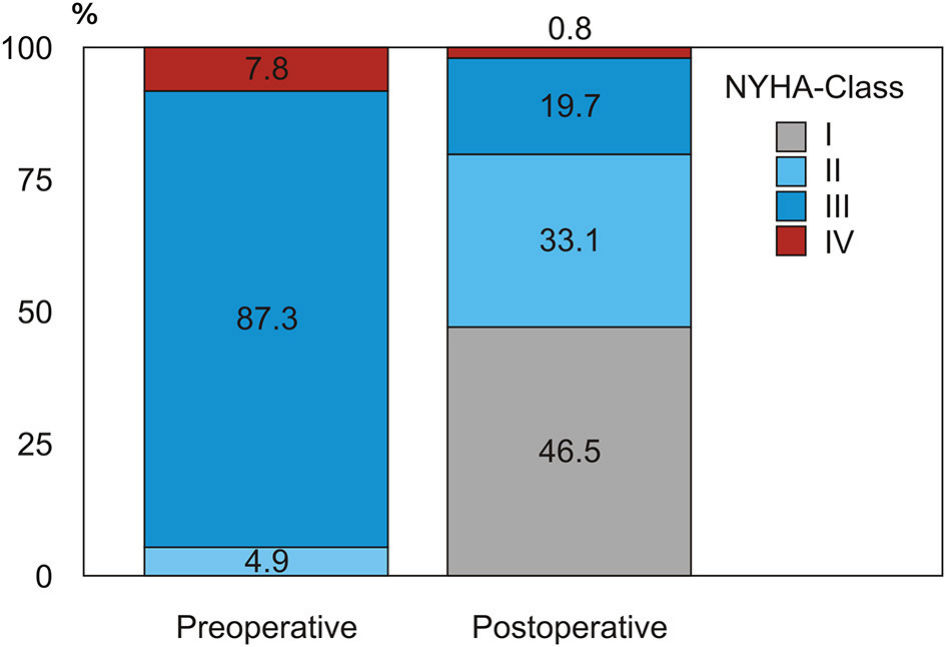
Redistribution of NYHA functional class before and after SVR in the surviving population.

During follow-up we observed a remarkable improvement in HF symptoms after SVR, with a positive redistribution of NYHA class in the whole population in the sense of a strong reduction of NYHA class III-IV quota after surgery in survivors (from 95.1% preoperatively to 20.5% in the early follow-up, *p* < 0.001) (Figure 4).

### Predictive Factors of 30-Day Mortality

Hospital and 30-day mortality were identical in our series. Thirteen of the 15 patients who died in hospital underwent combined SVR and CABG surgery, six underwent combined SVR, CABG and valvular surgery, and 10 required an intra-aortic balloon pump (IABP). Univariate logistic regression revealed factors potentially predictive of 30-day mortality: long CPB time and cross-clamp time, requirement of IABP or other temporary mechanical circulatory support systems, urgent surgery, postoperative sepsis and renal failure contributed to the higher 30-day mortality rate (Supplementary Table 1). Multivariate logistic regression showed that CPB time (*p* < 0.001) and postoperative LVEF (*p* < 0.001) were independent predictive factors of 30-day mortality. CPB time longer than 163 min was associated with an adverse outcome (sensitivity 0.765, specificity 0.789, AUC=0.890). Severity of preoperative HF, preoperative LVEF and CCT LV volumetric parameters did not play a substantial role in 30-day mortality.

### Late Mortality and Survival Determinants

Univariate logistic regression showed a potential predictive role in all-cause 5-year mortality of such factors as diabetes mellitus, peripheral artery disease, renal failure and atrial fibrillation. Preoperative MR ≥2+, preoperative LAVI, SVI, CI and postoperative LVEDVI, LVESVI and LAVI also had a potential predictive role in all-cause mortality (Supplementary Table 2). The multivariate regression analysis revealed that preoperative LVEDD and preoperative LAVI as well as patient age, presence of diabetes mellitus and renal failure were independent significant predictive factors of all adverse outcomes (Table 2).

**Table 2:** Multivariate logistic regression analysis for an adverse outcome (all-cause death, ventricular assist device or heart transplantation).

**Variable**	**Hazard ratio**	**95% Confidence interval**	***p-Value***
Preoperative LVEDD, mm	1.035	1.008–1.063	0.011
LAVI, ml/m^2^	1.023	1.008–1.038	0.003
Age at operation, y	1.032	1.002–1.063	0.039
Diabetes mellitus	2.223	1.27–3.9	0.039
Renal failure	2.3	1.2–4.4	0.012

*LVEDD, LV end diastolic diameter; LAVI, left atrium volume index*.

There was no difference in survival between men and women as shown by the Kaplan-Meier analysis (*p* = 0.593) and by the univariate regression analysis (male sex H*R* = 0.87; 95%CI 0.52–1.45).

### Changes in LV Geometry and Function

The changes in LV geometric and functional parameters measured by echocardiography and CCT after SVR are presented in Table 3.

**Table 3:** LV dimensions measured by echocardiography and in CCT before and after SVR.

**Parameter**	**Preoperative**	**Postoperative**	***p* Value**
**Echocardiography (*****n** **=*** **205)**			
LVEDD, mm	60.1 ± 9.95	55.1 ± 8.9	<0.001
LVEF, %	32.3 ± 10.6	39.5 ± 10.9	<0.001
MR, whole population	1.02 ± 0.8	0.36 ± 0.45	<0.001
MR, without MV surgery	0.79 ± 0.56	0.37 ± 0.44	<0.03
**CCT (*****n** **=*** **160)**	
LVEDVI, ml/m^2^	146 ± 52.4	97.3 ± 35.6	<0.001
LVESVI, ml/m^2^	100 ± 49.6	59.2 ± 33.4	<0.001
LAVI, ml/m^2^	60.7 ± 19.2	50.6 ± 18.7	<0.001
SVI, ml/m^2^	45.3 ± 11.7	37.6 ± 10.1	<0.001
LVEF, %	34.1 ± 12.1	43.1 ± 13.9	<0.001
CI, l/min/m^2^	3.08 ± 0.79	3.29 ± 0.75	0.022
LVSI, diastolic	0.40 ± 0.10	0.52 ± 0.18	<0.001
LVSI, systolic	0.31 ± 0.10	0.36 ± 0.17	<0.001

*LVEDD, LV end diastolic diameter; LVEF, LV ejection fraction; MR, mitral regurgitation; LVEDVI, LVESVI, LV end diastolic and end systolic volume index; LAVI, left atrium volume index; SVI, stroke volume index; CI, cardiac index; LVSI, LV sphericity index*.

LV volumetric parameters measured in CCT in 160 patients were significantly reduced after SVR: LVEDVI decreased about 33.0 ± 17.0% (*p* < 0.001) and LVESVI about 40.8 ± 21.5% (*p* < 0.001). LVEF measured in CCT increased significantly from 34.1 ± 12.1% to 43.1 ± 13.9% (*p* < 0.001) after SVR. The diastolic LV SI increased from 0.40 ± 0.10 to 0.52 ± 0.18 (*p* < 0.001); the systolic LV SI increased from 0.31 ± 0.10 to 0.36 ± 0.17 (*p* < 0.001).

### Survival Stratified by Preoperative LVESVI and Postoperative LVESVI

**Figure 5:**
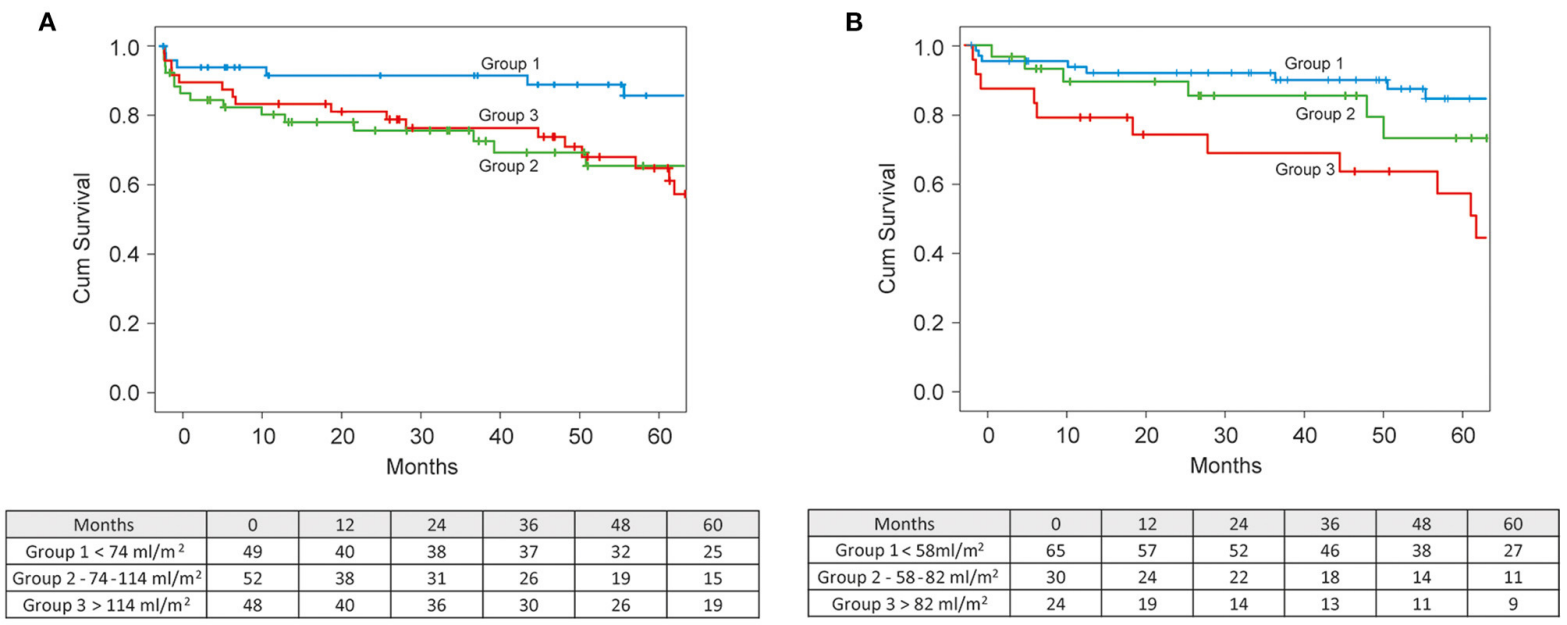
**(A)** Kaplan-Meier survival curves stratified by preoperative LVESVI. **(B)** Kaplan-Meier survival curves stratified by achieved postoperative LVESVI.

Survival stratification according to tertiles of preoperative LVESVI revealed a significantly higher survival rate in the lower tertile group (≤74 ml/m^2^) than in the groups with a LVESVI of 74–114 ml/m^2^ and ≥114 ml/m^2^ (*p* = 0.048). Five-year survival in the group with LVESVI ≤74 ml/m^2^ was 83.4% compared to 67.3 and 64.3% in the two other groups. An average 50 ml increase of preoperative LVESVI was associated with a 35% increase of the hazard of death (*p* = 0.043) (Figure 5A).

Survival stratification according to tertiles of the postoperative LVESVI demonstrated a significant difference in survival between tertiles (*p* = 0.016), with the lowest survival rate of 60.2% in the highest tertile of postoperative LVESVI (>66.2 ml/m^2^) compared to the survival rates of 84.6 and 85.3% in the middle (41.6–66.2 ml/m^2^) and lowest (<41.6 ml/m^2^) teriles, respectively (Figure 5B).

### Correlation Between Anticipated and Achieved Residual LV Volume and Estimated Aneurysm Volume

**Figure 6:**
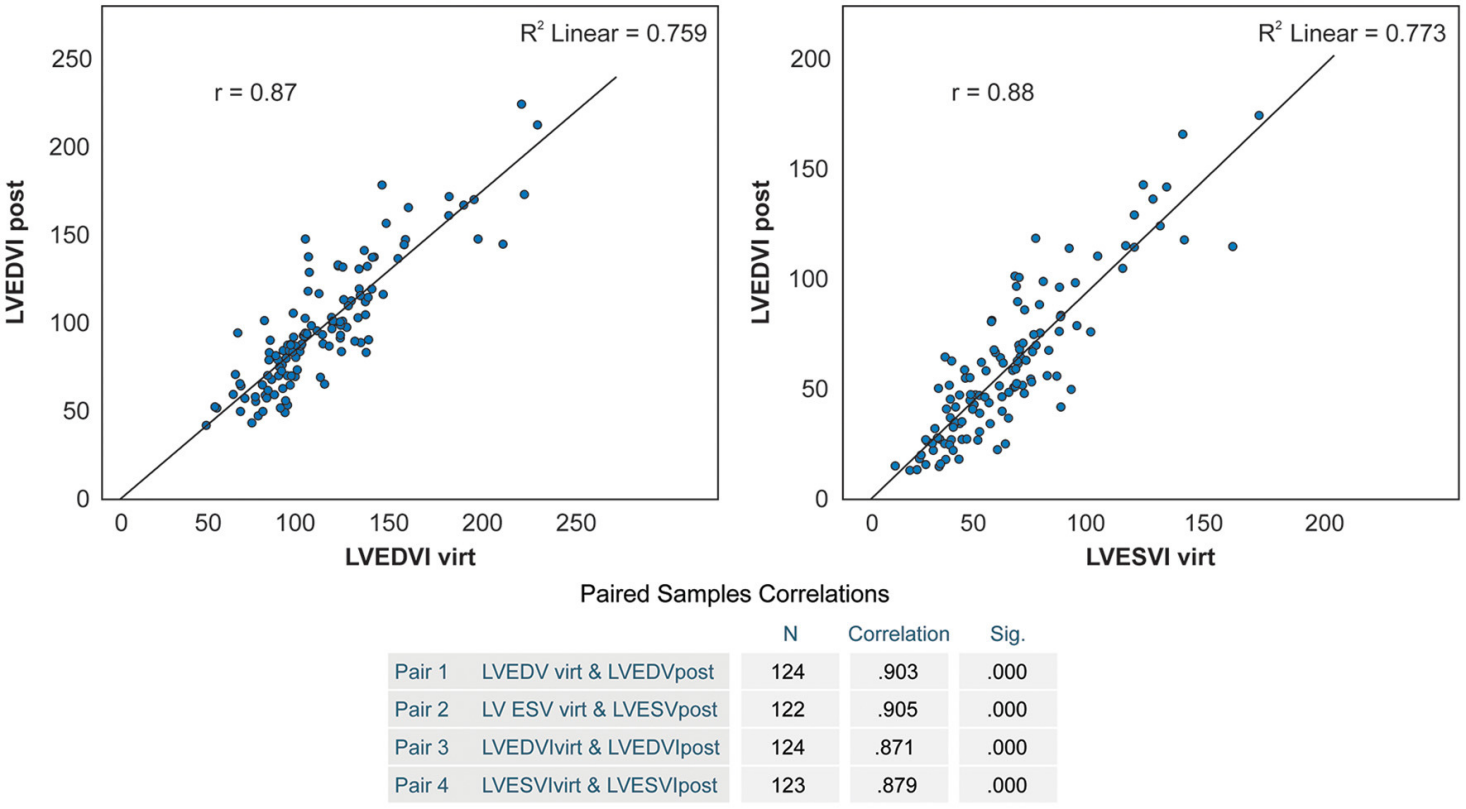
Correlation between anticipated and postoperatively achieved LVEDVI and LVESVI.

We observed a significant correlation between anticipated and effectively achieved postoperative LVEDV and LVESV (*r* = 0.903 and *r* = 0.904, respectively, *p* < 0.0001), and their indexed values (*r* = 0.87 and *r* = 0.88, respectively, *p* < 0.0001) (Figure 6). Anticipated LVEDVI was only 7.2 ± 18.0 ml/m^2^ higher than achieved LVEDVI (*p* = 0.00002), and anticipated LVESVI was only 2.3 ± 16.1 ml/m^2^ higher than achieved LVESVI (*p* = 0.109) (Table 4).

**Table 4:** Difference between anticipated and achieved postoperative LV Volumes.

**Parameter**	**Anticipated**	**Achieved**	**Mean difference**	***p-Value***
LVEDV, ml	203.3 ± 76.1	190.6 ± 78.5	12.7 ± 34.1	<0.001
LVESV, ml	119.9 ± 63.3	115.1 ± 70.7	4.8 ± 30.2	0.08
LVEDVI, ml/m^2^	104.6 ± 34.9	97.4 ± 35.9	7.2 ± 18	<0.001
LVESVI, ml/m^2^	61.6 ± 30.3	59.3 ± 33.7	2.3 ± 16.1	0.109

*LVEDV, LVESV, LV end diastolic and end systolic volume; LVEDVI, LVESVI, LV end diastolic and end systolic volume index diastolic and systolic*.

Mean estimated diastolic and systolic AnV were 88.5 ± 54.2 ml and 80.5 ± 56.4 ml, respectively. The relation of the estimated AnV to the actual preoperative LVEDV and LVESV was 28.9 ± 11.3% and 37.8 ± 14.7%, respectively. This relation closely matched with the effectively postoperative achieved relation of excluded part of LV volume to the whole LV volume—LVEDV by 33.1 ± 1.6% and LVESV by 41.2 ± 21.7%.

### Analysis of LV Segmental WMA

Aneurysms limited to seven antero-apical segments (1–7)—group 1—were associated with a lower death risk (*n* = 60, HR 0.52, CI 0.28–0.96, *p* = 0.038). The Kaplan-Meier analysis showed significantly better survival in these patients compared to all other patients (*p* = 0.035)—group 2 and 3—with a 5-year survival rate of 84.7% (95% CI 71.3–92.1) vs. 64% (95% CI 50.8–74.6) (Table 5).

**Table 5:** Five-year survival rates in defined subgroups of patients according to their LVA localization and extension.

**Group**	***N***	**5-year Survival**
**1** Antero-apical LVA (segments 7,8,13,14,15,16,17)	60	84.7 % (71.3–92.1)
**2** Antero-apical LVA + segment 1 or 9	9	62.2% (21.3–86.4)
**3** Antero-apical LVA + any other segments involved	85	63.7% (50.8–74.1)
**2+3** Groups	94	64 % (50.8–74.6)

*Group 1—antero-apical LVA (segments 7, 8, 13, 14, 15, 16, 17); Group 2—antero-apical LVA + segment 1 or 9; Group 3—antero-apical LVA + any other segments involved*.

*Bold values represents Group 1, Group 2, Group 3 and Group 2+3 respectively*.

**Figure 7:**
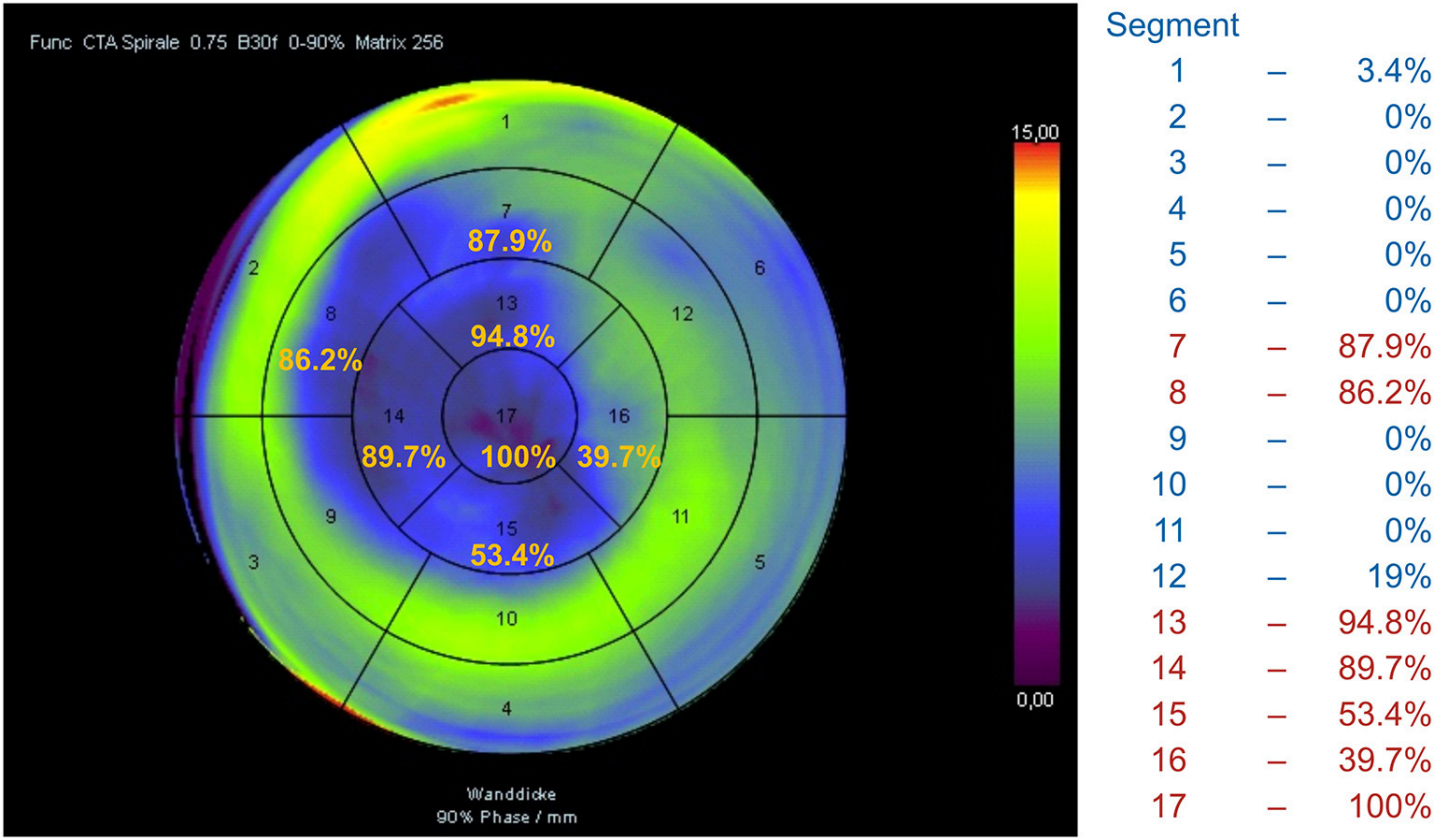
Automated cluster analysis based on the frequency of segment involvement allowed for identifying the scar pattern (Cluster 1) with a significantly better outcome compared to all other patients.

Furthermore, an automated cluster analysis based on the frequency of segment involvement and its impact on survival after SVR allowed for identifying the similar scar pattern (Cluster 1) with a significantly better outcome (Figure 7) compared to all other patients with HR 0.491 (0.263–0.916, *p* = 0.025) and a 5-year survival rate of 82.2% vs. 63.7% in all other patients (*p* = 0.022).

### Left Atrium Volume

LAVI decreased significantly from 60.7 ± 19.2 ml/m^2^ to 50.6 ± 18.7 ml/m^2^ (*p* < 0.001). Dividing by tertiles of preoperative LAVI revealed that patients in the lowest tertile (<49.5 ml/m^2^) had a lower risk of death than patients in the middle (49.5–67.6 ml/m^2^) and in the highest tertile (>67.6 ml/m^2^) of LAVI (long rank *p* = 0.005), with a 5-year survival in patients with LAVI <49.5 ml/m^2^ of 83.4% compared to the survival rate of patients in the middle and highest tertiles of 71.6 and 60.8%, respectively.

### Mitral Regurgitation

MR was reduced after surgery from 1.02 ± 0.80 to 0.36 ± 0.45 (*p* < 0.001) in the whole population and from 0.79 ± 0.56 to 0.37 ± 0.44 (*p* < 0.001) in patients without concomitant MV surgery.

### Role of LVSI

Both systolic and diastolic SI increased significantly in all patients after SVR (Table 3) due to a substantial shortening of the LV long axis in relation to the LV volume reduction. However, the multivariate regression analysis (χ^2^=1.7, *p* = 0.428) provided no evidence for an effect of pre-operative diastolic SI on survival.

## Discussion

This study is based on our experience of SVR performed between 2005 and 2016, since the modified Dor procedure was implemented as a standard technique in our institution.

The main results of the present analysis are that both CCT-derived preoperative and postoperative LVESVI are predictive for mid-term survival. The possibility to predict the volume reduction following SVR through the separation of the aneurysm volume using a commercially available CCT evaluation software tool allows for better therapy planning and patient selection. The CCT-based analysis of local WMA provides additional information for risk assessment: aneurysms limited to seven antero-apical segments were associated with a lower risk of mortality. The follow-up analysis shows a remarkable positive improvement in NYHA class after surgery as evidence for an improvement in HF symptoms.

### Diagnostic Testing

Accurate assessment of the LV geometrical distortion, aneurysm localization and extension, and viability of the remaining myocardium is decisive for a successful SVR (19, 20). The most advanced imaging modality with excellent standardized approaches for these purposes is cardiac magnetic resonance imaging (MRI) (19); however, an increasing number of patients with ischemic cardiomyopathy become carriers of implantable devices (in our cohort up to 40%), limiting the applicability of MRI.

Despite excellent spatial and temporal resolution, echocardiography depends highly on exact geometric alignment, patient anatomical characteristics and the operator's skills, and it often has limitations in the visualization of the apical region. Furthermore, 2D echocardiography only allows for calculating the ventricle volume based on the empiric approximated formula, and 3D real-time volume detection is not widespread or sufficiently validated. In this study we complementarily employed echocardiography to evaluate the LV diameters, systolic function and severity of MR. The profound analysis of further echocardiographic data in the represented patient cohort, especially the prognostic role of speckle-tracking echocardiography (LV global longitudinal and basal longitudinal strain), is presented separately (21).

Our reasons to use CCT for SVR planning were the possibility of exact true volume detection and geometrical analysis in primarily acquired three-dimensional data sets combined with a short examination time and lack of technical restrictions even in critical patients. Previous studies (4, 22) demonstrated that CCT is a valuable tool to evaluate LV and MV geometry and function. In our previous study (5) we showed that CCT data enable the precise analysis of LV volume, geometry and local WMA as well as the reliable recognition of aneurysm borders, thrombotic apposition and presence of LV pseudoaneurysm. This study focuses on prognostic important volumetric and functional characteristics and on the validation of the CCT-based separation of the aneurysm volume from the residual LV volume and thereby on improvement of the therapy targeting, with the further aim to avoid the restrictive residual LV volume.

However, we are well aware that CCT has clear limitations in detecting scar transmurality and viability of the remaining myocardium compared to MRI.

### Thirty-Day Mortality

In our series the overall 30-day mortality was 7.3%. Dor et al. (19) found hospital mortality rates of 8.1 and 4.8% in early and late series of patients who underwent SVR. Garatti et al. (23) reported a 30-day mortality of 8.3% in patients after SVR for anterior aneurysm. Jeganathan et al. (13) reported a hospital mortality of 13.3% in patients who underwent combined SVR and MV surgery. Their group identified NYHA class IV symptoms, preoperative atrial fibrillation, previous cardiac surgery and presence of ischemic MR as significant risk factors for increased hospital mortality. In our study, multivariate logistic regression showed that CPB time (*p* < 0.001) and postoperative LV EF (*p* < 0.001) but not the severity of preoperative HF, preoperative LV EF or CCT LV volume were independent predictive factors of 30-day mortality.

### Late Mortality and Survival Determinants

In our study population, 1- and 5-year actuarial survival rates were 82.7 and 72.1%, which is comparable to most published studies. A multivariate regression analysis revealed that preoperative LVEDD and LAVI as well as the patient's age, presence of diabetes mellitus and renal failure were independent significant predictive factors of all adverse outcomes (Table 2).

Efficient LV volume reduction toward a physiological range of a LVESVI of <60–70 ml/m^2^ is essential to improve survival after surgical repair. Di Donato et al. (7) showed that a postoperative LVESVI of >60 ml/m^2^ is an independent predictor of mortality. The analysis of the STICH trial data (2) showed that a statistically significant reduction in mortality was achieved only in patients attaining an LVESVI of <70 ml/m^2^. In our study group, both the preoperative and the postoperative LVESVI had a strong effect on survival. A cut-off point of ≤74 ml/m^2^ for preoperative LVESVI revealed the best 5-year survival rate of 86% (Figure 5A). An average 50 ml increase of preoperative LVESVI was associated with a 35% increase of the hazard of death (*p* = 0.043). A postoperative LVESVI of ≥82 ml/m^2^ was strongly predictive of all-cause mortality, with a lowest 5-year survival rate of 49% (Figure 5B).

The other important task is to avoid the restrictive residual LV volume after surgery. There were five patients with LVESVI <20 ml/m^2^ after surgery in our series, only one of them showed no improvement in HF symptoms during follow-up.

In this context, the possibility to predict the volume reduction using CCT volumetric tools is extremely valuable for better therapy planning and appropriate patient selection.

A CCT-based analysis of LV segmental WMA yielded additional valuable findings; it showed that the scar pattern typically involving seven antero-apical segments is associated with a better 5-year survival rate of 82.2% compared to 63.7% in all other patients with more segments involved (*p* = 0.022).

We observed a significant reduction of LAVI as immediate effect of SVR, probably through the improvement in LV diastolic function after SVR and/or in correlation with the reduction of MR, both in the whole population and in patients who underwent SVR only (Table 2). Preoperative LAVI was identified as an independent significant predictive factor of all adverse outcomes with a 5-year survival in patients with LAVI <49.5 ml/m^2^ of 83.4%, compared to a survival rate of 71.6 and 60.8% in patients in the middle and highest tertiles, respectively.

In our series both diastolic and systolic LVSI increased immediately after surgery due to a substantial shortening of the LV long axis in relation to LV volume reduction, but it is not clear whether this effect is necessarily negative. In the follow-up study of the STICH trial, Choi et al. (17) also reported a postoperative increase of the SI in the CABG+SVR group and an association of higher baseline SI with poorer postoperative survival both in CABG and CABG+SVR groups. In our series, multivariate proportional hazards regression modeling (χ2 = 1.7, *p* = 0.428) revealed no evidence for an effect of pre-operative diastolic SI on survival.

### Study Limitations

First, the study is subject to the usual limitations inherent to a retrospective analysis of prospectively collected data sets. Second, CCT imaging data were not available in all patients. Third, postoperative CCT data were obtained only several days after surgery and documented the immediate effects of SVR on LV volumes and function. However, they do not reflect the effects of subsequent ventricular reverse remodeling. Fourth, CCT-based assessment of local WMA as semiquantitative detection of akinetic or dyskinetic myocardial segments involved in an aneurysm or scar formation of other localizations is dependent on the operator's skills and only offers rough scar detection. Due to the high radiation burden, we did not consider the use of CCT-based viability assessment. The CCT-based strain analysis has clear limitations because of the low temporal resolution. The analysis of systolic and diastolic LV function, myocardial dyssynergy and viability based on a two-dimensional speckle-tracking echocardiography is not included in this study. Fifth, the provided CCT-based procedure to separate the aneurysm volume using a plane determined by three landmarks on the borders of scarred to intact LV myocardium and subsequent estimation of residual volumes using the commercially available CCT volumetric tool only allows for an approximate estimation of the possible volume reduction and does not incorporate the modeling of the surgical procedure in the sense of the creation of a neoapex.

## Conclusions

The results of this study suggest that CCT-guided SVR can be performed with good mid-term survival and significant relief of HF symptoms due to LV volume reduction, reverse remodeling and functional improvement. The modified Dor procedure is an adequate surgical approach to achieve the therapeutic goals of SVR. CCT as an alternative modality to MRI can deliver relevant data for surgical planning as well as important predictive parameters for adverse outcome. The provided possibility to predict the achievable volume reduction through the separation of the aneurysm volume using a commercially available CCT volumetric tool allows for better therapy planning and patient selection.

## Data Availability Statement

The data analyzed in this study is subject to the following licenses/restrictions: anonymized data. Requests to access these datasets should be directed to Natalia Solowjowa, solowjowa@dhzb.de.

## Ethics Statement

The study was performed according to the principles of the Declaration of Helsinki and approved by the ethics committee of the Charité – Universitätsmedizin Berlin (EA2/177/20). Written informed consent for surgery was obtained from all patients or their representatives.

## Author Contributions

NS performed the MSCT examinations, designed the study, analyzed and interpreted the data, and wrote the manuscript. ON and YH analyzed and interpreted the data and helped writing the manuscript. AM helped design the study, analyzed and interpreted the data, and helped writing the manuscript. FH and VF performed surgery and revised the manuscript critically for important intellectual content. CK performed surgery, conceived and designed the study, analyzed and interpreted the data, and wrote the manuscript. All authors contributed to the article and approved the submitted version.

## Funding

This work was supported by the DZHK (German Centre for Cardiovascular Research) and the BMBF (German Ministry of Education and Research).

## Conflict of Interest

VF disclosed financial relationships with the following entities: Medtronic GmbH, Biotronik SE & Co, Abbott GmbH & Co KG, Boston Scientific, Edwards Lifesciences, Berlin Heart, Novartis Pharma GmbH, JOTEC GmbH, and Zurich Heart. The remaining authors declare that the research was conducted in the absence of any commercial or financial relationships that could be construed as a potential conflict of interest.

## Publisher's Note

All claims expressed in this article are solely those of the authors and do not necessarily represent those of their affiliated organizations, or those of the publisher, the editors and the reviewers. Any product that may be evaluated in this article, or claim that may be made by its manufacturer, is not guaranteed or endorsed by the publisher.
