# Supplementary material for: Surgical Restoration of Antero-Apical Left Ventricular Aneurysms: Cardiac Computed Tomography for Therapy Planning
Source: Front Cardiovasc Med. 2022 Mar 28;9:763073. doi: 10.3389/fcvm.2022.763073 (PMC8996115; doi:10.3389/fcvm.2022.763073)
Supplement: Supplementary file 1 [file Data_Sheet_1.pdf]

Supplemental Material

Table S1. Univariate logistic regression analysis for predictors of 30-days mortality in the whole population.

Variable	Hazard Ratio	95% Confidence interval	*P Value*
CBP time	1.007	(1.004 - 1.010)	<0.001
Cross-clamp time	1.028	(1.015 - 0.040)	<0.001
IABP support	6.902	(2.4-19.7)	<0.001
LVAD support	10.567	(3.7-29.8)	<0.001
Emergency operation	6.04	(1.9-18.8)	<0.001
Postoperative EF Echo	0.91	(0.86 - 0.95)	<0.001
Previous CABG	3.55	(1.02-12.4)	0.034
Postop sepsis	8.25	(2.99-22.7)	<0.001
Postop renal failure	4.6	(1.04-20.2)	0.027

Table S2 Univariate logistic regression analysis for predictors of 5-years all-cause mortality in the whole population.

Variable	Hazard Ratio	95% Confidence interval	*P Value*
Age	0.95	[0.90;1.00]	0.044
Female sex	1.13	[0.67;1.90]	0.644
Diabetes mellitus	2.15	[1.33;3.47]	0.002
Arterial hypertension	1.74	[0.99;3.05]	0.053
Hypercholesterolemia	0.92	[0.56;1.53]	0.753
Peripheral artery disease	2.38	[1.27;4.45]	0.007
Renal failure	2.62	[1.51;4.52]	0.001
Atrial fibrillation	2.48	[1.38;4.48]	0.003
Preoperative echocardiography			
LV-EF	0.98	[0.95;1.00]	0.051
LV-EDD	1.02	[1.00;1.05]	0.042
Mitral regurgitation ≥ 2+	1.58	[1.20;2.07]	0.001
Postoperative echocardiography			
LV-EF	0.94	[0.91;0.96]	<0.001
LV-EDD	1.03	[1.00;1.05]	0.048
Mitral regurgitation ≥ 2+	1.49	[0.90;2.48]	0.119
Preoperative CT			
LV-EDVI, ml/m^2	1.00	[1.00;1.01]	0.313
LV-ESVI, ml/m^2	1.00	[1.00;1.01]	0.069
LV-ESVI/50, ml/m^2	1.35	1.001-1.82	0.042
LAVI, ml/m^2	1.02	[1.01;1.04]	<0.001
SVI, ml/m^2	0.96	[0.94;0.99]	0.002
LV-EF, %	0.96	[0.94;0.99]	0.004
CI, l/min/m^2	0.66	[0.44;0.97]	0.036

Postoperative CT			
LV-EDVI, ml/m^2	1.01	[1.01;1.02]	0.001
LV-ESVI, ml/m^2	1.02	[1.01;1.03]	<0.001
LAVI, ml/m^2	1.03	[1.01;1.05]	<0.001
SVI, ml/m^2	0.99	[0.96;1.03]	0.672
LV-EF, %	0.96	[0.94;0.99]	0.004
CI, l/min/m^2	1.09	[0.63;1.89]	0.764
Concomitant CABG	0.90	[0.52;1.55]	0.700
Concomitant MV repair	1.37	[0.77;2.43]	0.287
Patch repair	1.17	[0.59;2.31]	0.648
Non-patch repair	0.85	[0.43;1.68]	0.648
LV thrombectomy	1.49	[0.83;2.70]	0.183
Mean CBP time	1.01	[1.01;1.01]	<0.001
Cross-clamp time	1.01	[1.00;1.02]	0.013
IABP support	3.20	[1.91;5.39]	<0.001
LVAD support	21.4	[9.07;50.6]	<0.001
